# Clinical Tolerability and Safety of Ketogenic Diet in Patients with Gynecological Malignancies Undergoing Radiotherapy: Preliminary Results of a Prospective, Randomized, Open-Label Trial (KOMPARC)

**DOI:** 10.3390/nu18020312

**Published:** 2026-01-19

**Authors:** Marco Cintoni, Rosa Autorino, Raffaella Michela Rinaldi, Elena Leonardi, Marta Palombaro, Giuditta Chiloiro, Viola De Luca, Pauline Celine Raoul, Emanuele Rinninella, Esmeralda Capristo, Antonio Gasbarrini, Maria Antonietta Gambacorta, Maria Cristina Mele

**Affiliations:** 1 UOC di Nutrizione Clinica, Dipartimento di Scienze Mediche e Chirurgiche, Fondazione Policlinico Universitario A. Gemelli IRCCS, Largo A. Gemelli 8, 00168 Rome, Italy; elena.leonardi03@icatt.it (E.L.); marta.palombaro@guest.policlinicogemelli.it (M.P.); paulineceline.raoul@policlinicogemelli.it (P.C.R.); emanuele.rinninella@unicatt.it (E.R.); mariacristina.mele@unicatt.it (M.C.M.); 2 Centro di Ricerca e Formazione in Nutrizione Umana, Università Cattolica del Sacro Cuore, 00168 Rome, Italy; esmeralda.capristo@unicatt.it (E.C.); antonio.gasbarrini@unicatt.it (A.G.); 3 UOC Radioterapia, Dipartimento di Diagnostica per Immagini, Radioterapia Oncologica ed Ematologia, Fondazione Policlinico Universitario A. Gemelli IRCCS, Largo A. Gemelli 8, 00168 Rome, Italy; rosa.autorino@policlinicogemelli.it (R.A.); giuditta.chiloiro@policlinicogemelli.it (G.C.); viola.deluca@policlinicogemelli.it (V.D.L.); mariaantonietta.gambacorta@policlinicogemelli.it (M.A.G.); 4 Dipartimento di Medicina e Chirurgia Traslazionale, Università Cattolica del Sacro Cuore, 00168 Rome, Italy; 5 UOS Medicina della Grande Obesità, Dipartimento di Scienze Mediche e Chirurgiche, Fondazione Policlinico Universitario A. Gemelli IRCCS, Largo A. Gemelli 8, 00168 Rome, Italy; 6 UOC Medicina Interna e Gastroenterologia, Dipartimento di Scienze Mediche e Chirurgiche, Fondazione Policlinico Universitario A. Gemelli IRCCS, Largo A. Gemelli 8, 00168 Rome, Italy; 7 Dipartimento di Scienze Radiologiche ed Ematologiche, Università Cattolica del Sacro Cuore, 00168 Rome, Italy

**Keywords:** ketogenic diet, pelvic tumor, endometrial cancer, cervical cancer, radiotherapy

## Abstract

**Background**: Radiotherapy is a common treatment for gynecological malignancies, often accompanied by significant side effects that impact patient nutritional status. The ketogenic diet has been proposed as a complementary nutritional strategy to enhance treatment efficacy, manage side effects, and preserve body composition. However, its safety and feasibility in the oncological setting remain under-investigated. **Methods**: The KOMPARC study is a prospective, randomized controlled trial evaluating the adherence, safety, and clinical tolerability of a ketogenic diet versus a standard Mediterranean diet in patients with cervical and endometrial cancer undergoing radiotherapy. Before the start of the treatment, patients were randomized to either the ketogenic diet or the standard diet groups. Anthropometric measures, Hand Grip Test, and body composition parameters from bioimpedance analysis were taken before the start of treatment and at the end. Adherence, adverse events, and patient-reported outcomes were monitored throughout the treatment period. **Results**: A total of 33 patients were enrolled. Adherence rates were comparable between the KD and standard diet groups (46.1% vs. 25.0% interruption rate, *p* = 0.21). No significant differences were observed in the incidence of gastrointestinal toxicities (*p* = 0.56), diarrhea (*p* = 0.81), nausea (*p* = 0.94), or weight loss (*p* = 0.24). Both groups experienced significant weight reduction during therapy without differential loss of body cell mass or other body composition parameters. Quality of life assessments indicated varied symptom profiles, with the KD group reporting increased appetite loss and worry about weight. **Conclusions**: Preliminary findings suggest that the ketogenic diet is a safe and feasible nutritional intervention during radiotherapy for pelvic tumors. These results support further investigation into ketogenic dietary strategies as adjuncts in oncologic care.

## 1. Introduction

Cervical and endometrial cancers represent a significant global health burden, ranking among the most prevalent gynecological malignancies worldwide. Estimates from the International Agency for Research on Cancer’s (IARC) GLOBOCAN 2022 report cervical cancer as the fourth most common cancer among women globally, with an estimated 660,000 new cases and 350,000 deaths in 2022. Endometrial cancer also poses a substantial public health concern, with approximately 417,000 new cases and 97,000 deaths reported that same year globally, though its incidence varies by region [1]. The standard of care for these malignancies relies heavily on radiotherapy, either as a curative treatment—often combined with chemotherapy for locally advanced cervical cancer—or as an adjuvant therapy following surgery for endometrial carcinoma [1]. While effective, pelvic radiotherapy is frequently associated with substantial toxicity, particularly affecting the gastrointestinal and genitourinary tracts. These side effects can precipitate a decline in nutritional status, leading to weight loss and changes in body composition that may compromise treatment outcomes and quality of life. Consequently, there is a pressing clinical need for complementary nutritional strategies that can not only support patient nutritional status but also potentially enhance therapeutic efficacy and manage treatment-related toxicity [2].

In this context, the Ketogenic Diet (KD) has emerged as a promising metabolic intervention [3]. The KD, characterized by high-fat, very low-carbohydrate, and moderate protein intake, fosters a metabolic environment conducive to ketosis. The body transitions to using ketones, derived from fats, as its primary energy source, rather than glucose, which is a favored fuel for many cancer cells [4]. This metabolic reprogramming is proposed to induce stress on tumor cells, potentially inhibiting their growth and enhancing the efficacy of conventional treatments [5]. Beyond its potential anti-tumor effects, the KD has been investigated for its ability to preserve body composition during oncological treatments. Scientific literature has shown how the ketogenic diet can modulate tumor metabolism and sensitize cancer cells to chemotherapy and radiotherapy [6,7]. Building on this growing interest, several clinical investigations have explored the feasibility, safety, and potential impact of implementing a ketogenic diet alongside standard cancer therapies [8]. The KETOCOMP trials, including studies on breast, rectal, and head & neck cancer patients undergoing radiotherapy, have demonstrated the feasibility of achieving and maintaining nutritional ketosis in this patient population. For instance, Klement et al. (2020) reported that a KD based on natural foods was feasible in breast cancer patients undergoing curative radiotherapy, leading to significant reductions in body weight and fat mass while preserving skeletal muscle mass [9]. Similarly, in rectal cancer patients, the KD significantly reduced body weight and fat mass and showed a trend towards improved pathological tumor responses [10]. Furthermore, initial findings in head and neck cancer patients suggested that the KD might partially counteract the detrimental effects of chemoradiotherapy on body composition [11]. These studies consistently indicate that the ketogenic diet can be safely integrated into cancer treatment protocols, with patients generally tolerating the diet well and achieving sustained ketosis. The promising preliminary results from the KETOCOMP series, particularly regarding feasibility and body composition, provide a strong rationale for further investigation into the safety and adherence of a ketogenic diet in patients with gynecological malignancies undergoing radiotherapy. However, the safety and feasibility of this high-fat dietary approach, specifically in patients with gynecological malignancies—who are already prone to radiation-induced gastrointestinal distress—remain under-investigated.

The primary objective of the KOMPARC (Ketogenic Compliance in Patients Undergoing Radiotherapy for Pelvic Cancer) trial discussed in this paper is to rigorously assess the adherence and clinical tolerability of patients to the ketogenic diet during radiotherapy for pelvic cancers.

Thus, the main outcome of this preliminary data is assessing the safety outcomes and potential adverse events associated with the concurrent application of the ketogenic diet and radiotherapy in patients affected by gynecological cancer.

## 2. Materials and Methods

### 2.1. Study Design and Population

**Figure 1:**
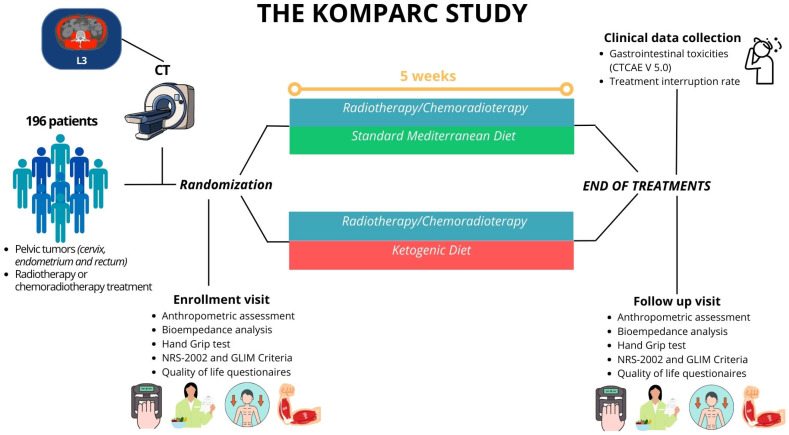
KOMPARC Study Protocol.

The KOMPARC study is an open-label, randomized controlled trial conducted at the Clinical Nutrition Unit of the “Policlinico Universitario Agostino Gemelli IRCCS”. The overall study design and timeline are illustrated in Figure 1.

Further details regarding the study protocol can be found on the ClinicalTrials.gov registry (ID: NCT05938322).

The estimated total study duration is 24 months from the ethics committee of the promoting center (CET Lazio Area 3, protocol ID 5022) approving the protocol, with a total of 196 planned patients to be enrolled.

The study population consists of patients with pelvic district neoplasms undergoing radiotherapy or chemoradiotherapy treatment. In particular, we focused on a subpopulation of patients with gynecologic malignancies, specifically cervical and endometrial cancers.

For endometrial cancer, standard treatment involves hysterectomy with bilateral salpingo-oophorectomy, possibly associated with lymph node assessment and followed, when indicated, by adjuvant chemotherapy and/or radiotherapy. Adjuvant external beam radiotherapy was delivered using volumetric modulated arc therapy (VMAT) to a total dose of 45 Gy (1.8 Gy/fraction), with possible concomitant or sequential boosts (up to 55–70 Gy) or brachytherapy (10–15 Gy over 2–3 weekly fractions) [12,13].

For locally advanced cervical cancer, exclusive chemoradiation (E-CT/RT) is the standard treatment. Radiotherapy was delivered to the tumor and regional nodes with a total dose of 45 Gy in 25 fractions over 5 weeks, using volumetric modulated arc therapy (VMAT) with daily image guidance. The involved lymph nodes received a simultaneous integrated boost (SIB) of 55 Gy (pelvic) or 57.5 Gy (aortic). Concurrent chemotherapy consisted of cisplatin alone. Within a week of external beam radiotherapy completion, high-dose-rate (HDR) brachytherapy. A total dose of 28 Gy in 4 fractions to HR-CTV and 14 Gy in 2 fractions to IR-CTV was delivered [13].

### 2.2. Inclusion and Exclusion Criteria

Patients were enrolled according to the following criteria.

Inclusion criteria for the study required patients to be (a) at least 18 years of age, (b) have a pelvic district neoplasm, (c) be undergoing radiotherapy treatment, and (d) provide a signed informed consent for personal data processing.

Patients were excluded if they were (a) younger than 18 or older than 75 years of age, (b) at risk of malnutrition based on NRS-2002 (NRS-2002 ≥ 3) or malnourished based on GLIM criteria, (c) receiving treatment for palliative purposes, or (d) had metastatic disease. Other exclusion criteria included (e) a diagnosis of diabetes mellitus, (f) pregnancy, or (g) breastfeeding. Patients with (h) food allergies that would prevent them from consuming the provided food, as well as those with (i) renal or (j) hepatic insufficiency, were also excluded.

### 2.3. Randomization and Dietary Intervention

Eligible patients were assigned to one of the two study groups using a computer-generated:

- Intervention Group (KD—Ketogenic Diet): Patients in this group will be prescribed a personalized variant of the classic ketogenic dietary plan, the Atkins Modified diet (MAD), characterized by the following macronutrient composition: carbohydrates <20g/day, proteins 1.2–1.5g/kg/day, and fats >70% of total caloric intake. The KD ratio, defined as the ratio of grams of fats to the sum of grams of protein and grams of carbohydrates, ranges from 1.5:1 to 1.8:1. This regimen will be followed throughout the entire radiotherapy treatment period.
- Control Group (SD—Standard Diet): Patients in this group will follow the Mediterranean diet model based on ESPEN guidelines, with a composition of 45–55% carbohydrates, 15–20% proteins, and 30–35% fats. This dietary plan will also be personalized and followed throughout the treatment.

Both groups will be given a food diary to complete for 2 days each week during the 5 weeks of treatment to analyze dietary intake and monitor adherence to the diet plan. To monitor ketosis, patients in the KD group utilized urinary ketone strips. We deliberately decided not to perform daily capillary blood ketone analysis to avoid imposing additional invasive procedures and burden on patients who were already undergoing daily radiotherapy sessions. A diet adherence rate of less than 50% of the prescribed days was classified as interrupted. This 50% cutoff was established to define a minimum biological exposure necessary to attribute potential physiological changes or adverse events to the specific dietary intervention. Consequently, the analysis of adherence/feasibility was performed on the total randomized population (33 patients), whereas the analysis of RT adverse effects, body composition, and patient-reported outcomes (PROs) was performed as a Per-Protocol analysis, evaluating only patients who maintained the minimum adherence threshold (28 patients).

### 2.4. Data Collection Procedures

The study employed a prospective design with two primary data collection points: a baseline assessment at Time 0 and a post-treatment assessment at Time 1.

At Time 0, upon enrollment and group assignment, patients provided detailed demographic and medical history data. A series of measures was also taken, including anthropometric evaluations (weight and height), a Bioelectrical Impedance Analysis (BIA) (BIA 101, Akern^®^, Florence, Italy), and a Hand Grip Test (DynX, Akern^®^, Florence, Italy). To assess PROs, participants completed the EORTC QLQ-C30 and EORTC QLQ-CAX24 quality of life questionnaires [14,15], as the QLQ-CAX24 questionnaire is considered an effective tool for monitoring changes in cachexia status during radiotherapy [16]. Additionally, malnutritional risk was assessed using the NRS-2002 [17], and malnutrition was diagnosed with the Global Leadership Initiative on Malnutrition (GLIM) criteria [18].

At Time 1, which was conducted at the conclusion of radiotherapy treatment, all procedures from the baseline assessment were repeated, except for the initial verification of criteria and the collection of demographic and historical data. In addition to these two visits, weekly phone calls were conducted throughout the treatment period to monitor adherence to the prescribed dietary plan and manage any potential side effects arising from the radiotherapy or chemoradiotherapy.

### 2.5. Study Objectives

The primary objective of the KOMPARC study was to evaluate patient adherence to the assigned diet treatment plan, specifically comparing adherence to the Standard Diet with adherence to the Ketogenic Diet. The secondary objectives included assessing the reduction of fat mass (FM) and the maintenance of body cell mass (BCM) in the KD group compared to the SD group, respectively. Additionally, the study aimed to assess patient tolerability to the treatment, measured by the percentage of patients who interrupted radiotherapy, and to investigate potential differences between the two groups in quality of life indices, including QLQ-C30 and QLQ-CAX24.

### 2.6. Statistical Analysis

The Kolmogorov-Smirnov test was used to evaluate whether the continuous variables had a normal distribution. Mean ± standard deviation was used to describe normally distributed variables, and number (percentage) was used to describe proportions. Student’s *t*-test or the Kruskal–Wallis test was used to compare continuous variables, according to the distribution. Comparisons of proportions were performed with the Chi-Square or Fisher’s exact test, where appropriate. Type I error was set at 0.05, and statistical significance was defined when *p* < 0.05 (two-tailed). A formal power calculation was conducted for the full KOMPARC trial (target patients: 196) to ensure adequate power for primary endpoints. However, no separate power calculation was performed for this preliminary interim analysis. Statistical analysis was performed using STATA (version 18.0).

## 3. Results

### 3.1. Baseline Data

**Figure 2:**
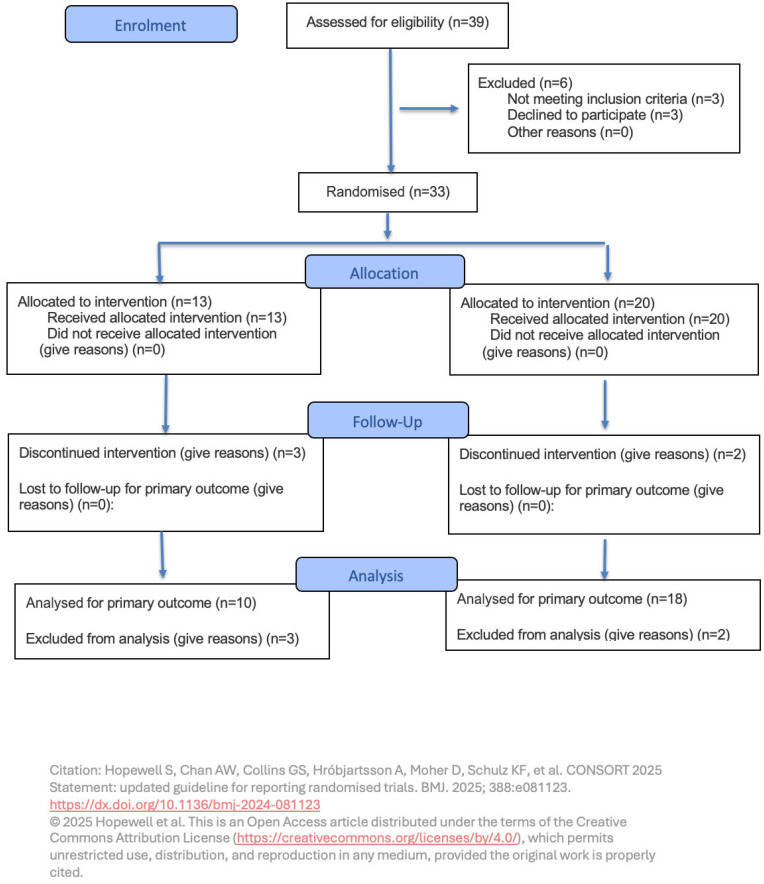
CONSORT 2025 Flow Diagram [19].

Thirty-three female patients were finally randomized in the trial after six patients were excluded from the initial cohort of 39 screened for eligibility for the following reasons: decline to participate (3), diabetes (2), and renal failure (1). Figure 2 resumes the enrollment process according to the CONSORT 2025 Flow Diagram.

No differences among the principal demographical and medical data at enrollment were found between the two analyzed groups (Table 1). Mean weight was 70.5 ± 16.2 kg, with a median of 68.5 kg (IQR: 55.8–82.0 kg). At baseline, there was a high prevalence of overweight and obesity, with 20 patients (60.6%) classified as overweight or obese (BMI ≥ 25 kg/m^2^) and 11 patients (33.3%) specifically as obese (BMI ≥ 30 kg/m^2^).

Of the enrolled patients, 17 (51.5%) had endometrial cancer, and 16 (48.5%) had cervical cancer. Within the endometrial cancer group, 7 (41.1%) were randomized in the KD group and 10 (58.8%) in the SD group. Similarly, of the 16 patients with cervical cancer, 6 (37.5%) were randomized in the KD group, while 10 (62.5%) were in the SD group.

At baseline, none of the patients were considered at risk of malnutrition based on an NRS-2002 score ≥ 3, and none were classified as malnourished by GLIM criteria.

### 3.2. Diet Interruption Rate and Adverse Effects

The overall diet interruption rate, calculated on the total study population, was 33.3% (11 patients). When comparing the two groups, six patients in the KD group (46.1%) and five patients in the SD group (25.0%) interrupted the diet (*p* = 0.21). Furthermore, dietary adherence varied between the groups. Patients in the KD group followed the diet for a mean of 15.3 ± 6.75 days, corresponding to an average adherence of 40.7%, while patients in the SD group maintained the diet for a mean of 20.6 ± 8.21 days, achieving a mean adherence of 54.2%. A total of 5 patients (3 in the ketogenic group; 2 in the standard group) were excluded from the final analysis because they did not meet the minimum adherence requirement of 50% (i.e., less than 18 out of 35 days) during the 5-week treatment period. One patient formally withdrew informed consent, while the remaining four patients (2 ketogenic; 2 standard) discontinued their participation due to non-adherence to the prescribed protocols, citing logistical difficulties with meal organization, portion control, and meal timing. Consequently, the final patient cohort for these specific analyses consisted of 10 patients in the ketogenic group (KD) and 18 patients in the standard group (SD).

Regarding Adverse Events (AEs), gastrointestinal toxicities were reported in 17 patients (60.7%) (Table 2). A breakdown of specific events shows that diarrhea occurred in 9 patients (32.1%), nausea in 8 patients (28.6%), and weight loss in 16 patients (57.1%). There was no statistically significant difference between the KD and SD groups for the overall incidence of gastrointestinal toxicities (*p* = 0.56), diarrhea (*p* = 0.81), nausea (*p* = 0.94), or weight loss (*p* = 0.24).

### 3.3. Body Composition Parameters

Significant weight changes were observed in both groups from baseline to the end of the study. The KD group experienced a decrease in weight from 75.1 ± 18.5 kg to 71.7 ± 16 kg (*p* = 0.03), while the SD group’s weight decreased from 66.6 ± 19.2 kg to 65.4 ± 18.9 kg (*p* = 0.02).

Three patients were at risk of malnutrition (NRS-2002 = 3) at the end of the study: 2 in the SD group and 1 in the KD group. Additionally, one patient in the SD group was diagnosed as malnourished according to GLIM Criteria.

No other significant changes were observed in anthropometric or body composition parameters in either group (Table 3).

### 3.4. Patients-Reported Outcomes (PROs)

Table 4 and Table 5 report the results of the QLQ-C30 and CAX-24 Questionnaires. Based on the questionnaire analysis, both groups experienced an increase in appetite loss (*p* = 0.02) and diarrhea (*p* = 0.01). In addition, the KD group showed a significant decrease in Global Health Status (*p* = 0.02), while the SD group experienced a substantial increase in fatigue (*p* = 0.01) and nausea and vomiting (*p* = 0.02).

From the CAX 24 questionnaire, both groups demonstrated a significant increase in food aversion (*p* = 0.03 for KD and *p* = 0.04 for SD) and dry mouth (*p* = 0.04 for KD and *p* = 0.03 for SD). Additionally, the KD group reported a significant increase in worry about eating and weight loss (*p* = 0.04), while the SD group showed a significant increase in eating difficulties (*p* = 0.02) and physical decline (*p* = 0.02).

## 4. Discussion

The KOMPARC study aims to analyze the adherence of patients with pelvic malignancies to a ketogenic diet (KD) during radiotherapy. Additionally, the study will examine the impact of the KD on adverse effects, treatment response, and survival rates. The primary objective of this preliminary analysis is to identify any early adverse effects. This will help us determine if the trial can proceed as outlined in the protocol or if adjustments to the intervention are needed.

Regarding adherence, although we found no statistically significant difference between the groups (*p* = 0.21), the absolute interruption rates (46.1% in KD vs. 25.0% in SD) differ noticeably. Given the limited sample size, it is unclear if this reflects a true disparity in tolerability. Therefore, these preliminary data need to be evaluated in the context of the global patient population to be enrolled in the full trial. This result challenges the common assumption that the restrictive nature of a ketogenic diet makes it exceptionally difficult to follow, particularly for patients already contending with the physical and psychological burdens of cancer treatment. Our data suggest that the challenge lies in maintaining adherence to any structured dietary plan during such treatment periods, regardless of its specific macronutrient composition, as noted by several authors who emphasize the critical role of personalized nutritional counseling and multidisciplinary support in improving patient compliance [20,21]. Previous studies have demonstrated that dietary adherence in cancer patients can vary widely but often improves with tailored guidance and ongoing support, suggesting that adherence strategies should not solely focus on dietary composition but also on psychological factors. Patient motivation, emotional state, and personal beliefs about the diet are all critical components that influence their ability to stick to a dietary regimen [22,23]. Andreyev et al. report that 80–90% of patients receiving pelvic radiotherapy experience acute gastrointestinal adverse effects. These adverse events are frequently linked to the tumor’s location and the volume of bowel irradiation, which affects the severity and presentation of symptoms. The most common AEs include diarrhea, proctitis, nausea, and vomiting [24].

This study provides evidence regarding the safety profile of a ketogenic diet in this vulnerable patient population. Global GI AEs were 70% (KD) and 56% (SD), with no statistically significant differences (*p* = 0.56), in the range of the described AEs. Moreover, no increase in nausea (*p* = 0.94), diarrhea (*p* = 0.81), GU toxicities (*p* = 0.27), or fatigue (*p* = 0.91) has been observed among patients of the two study groups. This observation is particularly reassuring given that these toxicities are common side effects of pelvic radiotherapy, as previously indicated in relevant literature [25,26]. The absence of a negative impact on these parameters suggests that the ketogenic diet does not exacerbate the radiation-induced inflammatory response in the gut mucosa, supporting findings that indicate ketogenic diets may positively modulate the body’s inflammatory markers [27].

Patient-reported outcomes are important to describe the impact of medical treatments on the quality of life. By examining significant differences in QoL between the KD and SD groups, this study contributes to the growing body of literature employing validated questionnaires such as the EORTC QLQ-C30 and CAX24. The KD group’s notable decrease in Global Health Status, alongside increased instances of appetite loss and diarrhea, reflects the complex interplay between dietary interventions and the challenges faced by patients undergoing cancer treatment. Comparable findings have emerged in studies utilizing the EORTC QLQ-C30, indicating that cancer treatment can lead to fluctuations in patients’ global health and symptom profiles due to the disease itself and its therapies [28,29] Specific to our findings, prior research has reported that cancer patients often experience increased fatigue and nausea during treatment, which are significant symptoms also observed in the SD group of our study [30,31]. Similar patterns were noted where patients on standard dietary regimens reported higher instances of eating difficulties and physical decline, corroborating the notion that dietary composition may influence but is not solely responsible for the patient’s overall quality of life and symptomatology [32,33,34,35]. The increase in worries related to eating and weight loss observed in the KD group might align with literature discussing the psychological burdens associated with restrictive diets in a cancer context [34,35]. This significant decline in Global Health Status and the heightened anxiety regarding eating and weight loss observed in the KD group underscore the substantial psychological burden imposed by this restrictive dietary regimen. Implementing a radical change in dietary habits during the stress of cancer treatment appears to have negatively impacted patient well-being, likely due to the loss of “comfort foods” and the social isolation that can accompany strict dietary adherence. To mitigate these adverse psychological effects, future interventions must go beyond standard nutritional education to include dedicated nutritional counseling. This support is essential to help patients distinguish between healthy diet-induced weight loss and pathological cachexia, thereby reducing anxiety and improving overall QoL. Moreover, the examination of food aversion and dry mouth in both groups provides insights into the broader implications of dietary adherence, as similar sensations of discomfort related to dietary intake have been documented in previous studies investigating the impacts of treatment on patient quality of life [36,37]. Such reports suggest that regardless of dietary strategy employed, cancer patients frequently navigate a battleground of gastrointestinal and psychological challenges.

Another crucial finding relates to the observed weight loss. While a statistically significant decrease in body weight was detected in both groups, this reduction must be contextualized within the baseline characteristics of the cohort, where 60.6% of patients were overweight or obese. Importantly, this weight loss appears to be clinically non-detrimental, as it was not accompanied by a significant reduction in BCM or FFMI in either group. The preservation of lean tissue suggests that the weight reduction was primarily driven by loss of adipose tissue rather than the skeletal muscle depletion characteristic of cancer cachexia. Consequently, in this specific population of overweight patients with pelvic malignancies, the dietary intervention supported weight management without exacerbating the risk of sarcopenia or malnutrition. This strongly suggests that the weight loss is primarily a consequence of the disease-induced catabolic state and the systemic effects of treatment, rather than a specific outcome of the ketogenic dietary intervention [38]. The fact that the ketogenic diet did not lead to an accelerated or disproportionate loss of body weight compared to the standard diet serves as a key safety indicator [39]. Notably, a large proportion of our patient cohort was either overweight or obese at baseline. Future studies should investigate whether a moderate weight loss in this population could have a positive impact on the overall inflammatory status. Additional studies have shown that similar dietary interventions typically lead to weight stabilization rather than excessive loss, aligning with our findings of comparable changes in body composition parameters across both groups. Specifically, the ketogenic diet did not appear to preferentially deplete BCM, which is a major concern in oncology nutrition due to its correlation with functional decline and poorer prognosis [40].

While these preliminary findings are promising, several limitations must be acknowledged. The reduced sample size, particularly the final cohort of only ten patients in the KD arm, is a significant limitation, rendering the study underpowered to detect smaller effect sizes or to perform subgroup analyses. Consequently, the fragmentation of the cohort across two distinct tumor types (cervical and endometrial) precluded any statistical comparison between these subgroups, despite their differing clinical profiles. Therefore, these results should be interpreted as exploratory and primarily indicative of safety and feasibility rather than efficacy. Although stratified analyses were contemplated to examine how these disease-specific factors impacted adherence and adverse events, they were not performed due to the limited sample size. Consequently, the pooled analysis may obscure specific challenges faced by patients undergoing more intensive combined-modality treatments. Future analyses in the fully recruited cohort will be stratified by tumor type to definitively assess these disease-specific impacts. This study deliberately selected a cohort of patients with gynecological malignancies, partially due to their typically younger age and presumed higher compliance potential compared to the general pelvic radiation population. Additionally, since this is a preliminary report, there’s a lack of a long-term follow-up period, which prevents us from concluding the diet’s long-term safety, efficacy, or whether its effects persist after the intervention ends. Another limitation of this study is the reliance on urine ketone levels rather than blood ketone measurements. While capillary blood testing is the gold standard for assessing ketogenesis [41], we opted for urine monitoring to minimize invasiveness and patient distress during the intensive radiotherapy course. Furthermore, the safety analysis primarily focused on radiotherapy-related toxicities using standard CTCAE v5.0 criteria. Consequently, specific symptoms associated with the initial metabolic transition (commonly termed “Keto Flu”), such as headache, dizziness, and muscle cramps, were not systematically monitored as distinct endpoints. While key overlapping symptoms such as fatigue and nausea were tracked, the study design prevents a specific assessment of the “Keto Flu” burden distinct from radiation-induced side effects.

Some of these limitations, inherent to preliminary studies, will be directly addressed in the planned larger randomized controlled trial, which is designed to include a larger cohort of participants and an extended follow-up period. This study aims to provide a more comprehensive understanding of the ketogenic diet’s potential therapeutic benefits while ensuring that the methodology employed will be robust enough to detect significant differences in patient outcomes.

## 5. Conclusions

Based on these preliminary observations, KD appears to be a feasible nutritional strategy for patients with gynecological malignancies during the acute phase of radiotherapy, provided that adequate adherence support is available. While the diet did not significantly increase treatment-related gastrointestinal toxicities or reduce muscle mass, it was associated with specific quality-of-life trade-offs, including increased appetite loss and anxiety regarding weight reduction. Therefore, while the intervention appears safe in the short term, its implementation requires careful nutritional monitoring. Ultimately, these exploratory findings provide the necessary safety rationale to proceed with the full KOMPARC trial recruitment, which is needed to definitively assess the diet’s impact on tumor control and long-term survival outcomes.

## Figures and Tables

**Table 1:** Principal Demographic and Medical Data at Enrollment Visit.

	Total (33 Patients)	KD Group (13 Patients)	SD Group (20 Patients)	*p*
Female Sex	33 (100%)	13 (100%)	20 (100%)	-
Adjuvant Chemotherapy	22 (66.7%)	8 (61.5%)	14 (70.0%)	0.58
Endometrium	17 (51.5%)	7 (41.1% of endometrial cancer)	10 (58.8% of endometrial cancer)	0.82
Cervix	16 (48.5%)	6 (37.5% of cervical cancer)	10 (62.5% of cervical cancer)	0.82
Weight	70.5 ± 16.2	73.3 ± 15.3	69.2 ± 17.1	0.49
Height	1.59 ± 0.06	1.58 ± 0.05	1.59 ± 0.07	0.44
BMI	28.0 ± 6.8	29.3 ± 6.4	27.1 ± 7.0	0.37
Obesity (BMI ≥ 30 kg/m^2^)	11 (33.3%)	5 (38.5%)	6 (30.0%)	0.61
Overweight/Obesity (BMI ≥ 25 kg/m^2^)	20 (60.6%)	9 (69.2%)	11 (55.0%)	0.41
Hand Grip T0	20.7 ± 5.2	19.4 ± 3.9	21.7 ± 5.9	0.23
Rz T0	548 ± 88	537 ± 79	554 ± 93	0.59
Xc T0	55.2 ± 8.3	54.8 ± 6.9	55.5 ± 9.3	0.81
PhA	5.75 ± 0.76	5.72 ± 0.68	5.77 ± 0.83	0.87
SPhA	−0.24 ± 0.88	−0.24 ± 0.79	−0.23 ± 0.94	0.98
BCM%	52.5 ± 3.9	52.9 ± 3.5	52.2 ± 4.3	0.63
FM%	32.0 ± 9.5	34.3 ± 9.3	30.5 ± 9.6	0.27
FFMI	26.9 ± 4.0	26.3 ± 4.1	27.2 ± 4.0	0.54
TBW%	49.7 ± 6.9	48.1 ± 6.7	50. ±6.9	0.30
BMR T0	1468 ± 139	1472 ± 107	1465 ± 160	0.90

Abbreviations: BMI: Body Mass Index; Rz: Resistance; Xc: Reactance; PhA: Phase Angle; SPhA: Standardized Phase Angle; BCM: Body Cell Mass; FM: Fat Mass; FFMI: Fat Free Mass Index; TBW: Total Body Water; BMR: Basal Metabolic Rate.

**Table 2:** Treatment-related adverse events (according to CTCAE v5.0).

	Total (28 Patients)	KD Group (10 Patients)	SD Group (18 Patients)	*p*
Gastrointestinal	17 (60.7%)	7 (70.0%)	10 (55.6%)	0.56
Diarrhoea	9 (32.1%)	4 (40.0%)	5 (27.8%)	0.81
Nausea	8 (28.6%)	3 (30.0%)	5 (27.8%)	0.94
Genitourinary	3 (10.8%)	2 (20.0%)	1 (5.5%)	0.27
Fatigue	11 (39.2%)	4 (40.0%)	7 (38.9%)	0.91
Weight loss	16 (57.1%)	7 (87.5%)	9 (64.3%)	0.24

**Table 3:** Differences in body composition parameters between the two groups at baseline and after treatment.

	KD Group (10 Patients)			SD Group (18 Patients)		
	Enrollment	End-of-Treatment	*p*	Enrollment	End-of-Treatment	*p*
Weight	75.1 ± 18.5	71.7 ± 16	0.03	66.6 ± 19.2	65.4 ± 18.9	0.02
Hand Grip	18.4 ± 3.0	19.0 ± 4.0	0.52	22.2 ± 6.7	21.6 ± 7.4	0.64
Rz	541 ± 66	543 ± 68	0.85	559 ± 102	552 ± 109	0.39
Xc	54.5 ± 8.2	53.6 ± 7.2	0.6	55.9 ± 9.7	53.5 ± 11.0	0.11
PhA	5.78 ± 0.64	5.63 ± 0.27	0.37	5.79 ± 0.90	5.63 ± 0.89	0.13
BCM%	51.9 ± 3.1	51.6 ± 1.5	0.79	52.3 ± 4.7	51.5 ± 5.0	0.16
FM%	37.9 ± 10.7	36.2 ± 9.5	0.22	28.6 ± 10.0	27.3 ± 10.1	0.09
FFMI	25.4 ± 4.4	26.1 ± 3.8	0.23	28.0 ± 4.3	28.5 ± 4.6	0.06
TBW	45.5 ± 7.8	46.7 ± 6.8	0.21	52.1 ± 7.2	53.2 ± 7.3	0.06
BMR	1455 ± 90	1253 ± 532	0.32	1470 ± 182	1461 ± 185	0.28
MQI BIA	0.96 ± 0.19	0.83 ± 0.35	0.14	1.11 ± 0.25	1.06 ± 0.21	0.41

Rz: Resistance; Xc: Reactance; PhA: Phase Angle; SPhA: BCM: Body Cell Mass; FM: Fat Mass; FFMI: Fat Free Mass Index; TBW: Total Body Water; BMR: Basal Metabolic Rate.

**Table 4:** Changes in quality-of-life scores (QLQ-C30) from baseline to post-treatment.

	KD Group (10 Patients)			SD Group (18 Patients)		
	Enrollment	End-of-Treatment	*p*	Enrollment	End-of-Treatment	*p*
Global health status	58.3 (50.0–75.0)	62.5 (29.2–70.8)	0.02	75 (66.7–83.3)	66.7 (54.2–83.3)	0.36
Physical functioning	90.0 (73.3–93.3)	62.5 (29.2–70.8)	0.32	86.7 (80–93.3)	66.7 (54.2–83.3)	0.052
Role functioning	75.0 (33.3–100)	75 (25–100)	0.51	91.7 (66.7–100.0)	66.7 (66.7–100)	0.11
Emotional functioning	75.0 (66.6–75.0)	75.0 (54.2–100.0)	0.52	66.7 (50.0–91.7)	75.0 (66.7–100.0)	0.71
Cognitive functioning	83.3 (83.3–100.0)	75.0 (66.7–100.0)	0.52	91.7 (66.7–100)	91.7 (75.0–100.0)	0.58
Social functioning	66.7 (66.7–100.0)	75.0 (66.7–100.0)	0.66	100.0 (83.3–100.0)	75.0 (66.7–100.0)	0.17
Fatigue	72.2 (33.3–100.0)	41.7 (27.8–66.7)	0.10	22.2 (11.1–33.3)	33.3 (22.2–44.4)	0.01
Nausea and vomiting	0 (0–50)	25.0 (8.3–41.7)	0.66	0 (0–0)	8.3 (0.0–33.3)	0.02
Pain	33.3 (33.3–66.7)	8.3 (0.0–50.0)	0.62	0.0 (0.0–16.7)	16.7 (0.0–33.3)	0.16
Dyspnoea	33.3 (0.0–100.0)	33.3 (0.0–66.7)	0.94	0 (0–0)	0.0 (0.0–33.3)	0.65
Insomnia	50 (0–100)	33.3 (16.7–50.0)	0.23	0.0 (0.0–33.3)	33.3 (0.0–50.0)	0.057
Appetite loss	0.0 (0.0–33.3)	66.7 (33.3–66.7)	0.02	0 (0–0)	0.0 (0.0–33.3)	0.02
Constipation	0 (0–0)	0 (0–0)	0.92	0.0 (0.0–33.3)	0.0 (0.0–33.3)	0.38
Diarrhoea	0.0 (0.0–33.3)	50.0 (33.3–100.0)	0.02	0 (0–0)	33.3 (33.3–33.3)	0.01
Financial difficulties	33.3 (0.0–66.7)	33.3 (0.0–66.7)	0.56	16.7 (0.0–33.3)	33.3 (16.7–33.3)	0.35

**Table 5:** Changes in quality-of-life scores (CAX-24) from baseline to post-treatment.

	KD Group (10 Patients)			SD Group (18 Patients)		
	Enrollment	End-of-Treatment	*p*	Enrollment	End-of-Treatment	*p*
Food aversion	6.7 (0.0–20.0)	23.3 (16.7–43.3)	0.03	0.0 (0.0–6.7)	6.7 (0.0–20.0)	0.04
Eating & weight loss worry	0 (0–0)	0.83 (0.0–22.2)	0.04	0.0 (0.0–11.1)	0 (0–0)	0.30
Eating difficulties	0 (0.0–11.1)	11.1 (0.8–33.3)	0.07	0 (0–0)	0.0 (0.0–16.7)	0.02
Loss of control	22.2 (11.1–22.2)	25 (3.4–33.3)	0.29	11.1 (5.6–22.2)	11.1 (5.6–33.3)	0.42
Physical decline	0 (0–0)	11.1 (5.6–33.3)	0.07	0 (0–0)	0.0 (0.0–22.2)	0.02
Dry mouth	0.0 (0.0–33.3)	33.3 (33.3–83.3)	0.04	0.0 (0.0–33.3)	33.3 (0.0–33.3)	0.03
Indigestion	0 (0–0)	0.0 (0.0–50.0)	0.15	0 (0–0)	0.0 (0.0–33.3)	0.08
Forced to eat	0 (0–0)	0 (0–0)	-	0 (0–0)	0 (0–0)	-
Information	66.7 (33.3–66.7)	66.7 (66.7–100.0)	0.20	66.7 (33.3–66.7)	66.7 (66.7–100.0)	0.13

## Data Availability

The original contributions presented in this study are included in the article. Further inquiries can be directed to the corresponding authors.
